# Ultimately Bounded Filtering for Time-Delayed Nonlinear Stochastic Systems with Uniform Quantizations under Random Access Protocol

**DOI:** 10.3390/s20154134

**Published:** 2020-07-25

**Authors:** Jiyue Guo, Zidong Wang, Lei Zou, Zhongyi Zhao

**Affiliations:** 1 College of Electrical Engineering and Automation, Shandong University of Science and Technology, Qingdao 266590, China; guojy_sdust@163.com (J.G.); zhaozy_sdust@163.com (Z.Z.); 2 Department of Computer Science, Brunel University London, Uxbridge UB8 3PH, Middlesex, UK; zouleicup@gmail.com

**Keywords:** uniform quantization, random access protocol, ultimately bounded filtering, discrete-time systems, time-delays

## Abstract

This paper investigates the ultimately bounded filtering problem for a kind of time-delay nonlinear stochastic systems with random access protocol (RAP) and uniform quantization effects (UQEs). In order to reduce the occurrence of data conflicts, the RAP is employed to regulate the information transmissions over the shared communication channel. The scheduling behavior of the RAP is characterized by a Markov chain with known transition probabilities. On the other hand, the measurement outputs are quantized by the uniform quantizer before being transmitted via the communication channel. The objective of this paper is to devise a nonlinear filter such that, in the simultaneous presence of RAP and UQEs, the filtering error dynamics is exponentially ultimately bounded in mean square (EUBMS). By resorting to the stochastic analysis technique and the Lyapunov stability theory, sufficient conditions are obtained under which the desired nonlinear filter exists, and then the filter design algorithm is presented. At last, two simulation examples are given to validate the proposed filtering strategy.

## 1. Introduction

Owing to their great significance in signal processing and control applications, filtering problems have gradually become a mainstream topic of research in recent years. The primary idea of the filtering problem is to reconstruct the immeasurable state variables of an underlying plant based on the noisy measurements. The past several decades have witnessed a surge of research enthusiasm towards developing various filtering algorithms, and a great many representative works have been included in the literature, see, e.g., [1,2,3,4,5,6,7,8,9,10]. Generally speaking, the filtering strategies existing in the literature mainly include the $H_{\infty }$ filtering [8,11], ultimately bounded filtering [12,13,14,15], optimal filtering [16,17,18,19,20], and variance-constrained filtering [21]. Among others, the ultimately bounded filtering strategy has been found particularly suitable to handle the filtering issue of time-invariant systems with guaranteed steady-state filtering performance.

It has been well recognized that the phenomenon of time-delays is frequently found in various industrial plants such as networked systems, chemical systems, and biological systems. Such a phenomenon, if not addressed properly, is likely to incur performance deteriorations or even system instability. Hence, much research effort has been directed towards the analysis/design problems concerning filtering issues with time delays in the past few decades. Up to now, there have been roughly four kinds of time delays available in the existing literature, i.e., time-varying delays, discrete delays, distributed delays, and mixed delays, see, e.g., [6,18,22]. Recently, the filter design problem for time-delay nonlinear stochastic systems has stirred considerable research interest because of its great significance in both theoretical and practical aspects, see, e.g., [6,23,24]. In particular, the filtering issue has been dealt with in [24] for time-delayed stochastic systems with nonlinearities, where a variance-constrained approach has been applied to design the relevant parameters.

Along with the rapid development and wide application of the network-based communication technique, the networked systems have been capturing constant research attention in the last two decades [25]. In a networked control system, the information transmissions between different system components are implemented via the shared network channels rather than the traditional point-to-point cabling. As compared with traditional non-networked systems, the utilization of the communication network brings many advertised merits which include, but are not limited to, more flexible installation and lower installation and maintenance costs. These advantages have paved the way for the popularity of networked systems in a wide range of domains. Nevertheless, great challenges have also been posed for engineers and scientists due to the inevitable network-induced phenomena, and some typical issues include packet dropouts, communication delays, fading channels, quantization effects, and nonuniform sampling. Such networked-induced phenomena, if not well addressed, are likely to deteriorate the system performance. Consequently, it is quite necessary to give full consideration to the networked-induced phenomena in the course of designing controllers/filters for networked systems, and some excellent results have been published in the recent literature, see, e.g., [5,6,9,26,27,28,29,30,31].

Among a variety of networked-induced features, quantization is deemed to be one of the most important factors that affect the performance of networked systems. In practical engineering, it is often the case that the signals might be quantized before transmitted via the communication channel, which would give rise to certain “quantization error" and the degraded control/filtering performance [32]. So far, the filtering problem subject to quantization effects has gained a notably growing research interest, see for instance [33,34,35] and the references therein. Roughly speaking, two types of modeling approaches have appeared for the quantization in existing literature: one is the uniform quantization model corresponding to the fixed-point quantization phenomenon, and the other is the logarithmic quantization model corresponding to the floating-point quantization phenomenon [5,36].

In a networked system with multiple sensors, it is usually unrealistic to assume that all the sensors are simultaneously granted access to the communication channel to send the measurement signals [37]. Obviously, the simultaneous data transmissions through a shared communication channel with limited bandwidth could result in severe data collisions and other networked-induced phenomena. As such, different scheduling protocols have been proposed to govern the order of the sensors by allocating the network-access-opportunity at each transmission instant according to certain agreements. There are three sorts of frequently used scheduling protocols in practice, i.e., the Round-Robin protocol [6,38,39,40,41], the Try-Once-Discard protocol [42,43] and the Random Access protocol (RAP) [7,8,22,44]. From the perspective of industry application, the RAP is the most preferred one due to its simplicity and extensibility. Based on the scheduling principle of the RAP, the network nodes would obtain their network access privileges in a random manner.

Concluding the aforementioned discussion, it seems interesting to investigate the ultimately bounded filtering problem for a kind of time-delay nonlinear stochastic systems with RAP and uniform quantization effects (UQEs) due to its practical significance and theoretical importance. This is definitely a non-trivial task because of the following technical difficulties: (1) how to develop a method to compute the ultimate bound of the filtering error? and (2) how to devise sufficient conditions under which the desired filter exists? As such, the primary objective of this paper is to provide adequate responses to the above two questions. The main novelties of this paper are outlined as follows: (1) for the first time, the ultimately bounded filtering problem is examined for a kind of time-delay nonlinear stochastic systems under RAP and UQEs; (2) a theoretical framework is built to analyze the ultimate boundedness of the filtering error by utilizing the stochastic analysis technique; and (3) the filter gain matrices are given by resorting to the feasibility of a few linear matrix inequalities (LMIs).

The remainder of this paper is outlined as follows. In Section 2, the ultimately bounded filtering problem is stated for the networked systems with RAP and UQEs. In Section 3, some sufficient conditions are established, based on the standard theoretical analysis, to guarantee the ultimately bounded performance of the designed filter. Section 4 provides two simulation examples to verify the feasibility of the proposed filtering scheme. Finally, the conclusion is given in Section 5.

Notations: In this paper, $R^{n}$ and $R^{n\times m}$ represent, respectively, the *n*-dimensional Euclidean space and the set of all n×m real matrices. ∥δ∥ and ${∥\delta ∥}_{\infty }$ stand for, respectively, the Euclidean norm and the infinite norm of a vector δ. For real symmetric matrices *X* and *Y*, X≤Y (X<Y) indicates that X−Y is negative semi-definite (negative definite). For a matrix *B*, $B^{T}$ and tr{B} refer to its transpose and trace, respectively. ${\left[B\right]}_{n\times m}$ represents the set of all n×m real matrices. ∥B∥ denotes the spectral norm of the matrix *B*. $\lambda_{\min}\left(B\right)$ denotes the minimum eigenvalue of *B*. For a random event “·”, Prob{·} represents the occurrence probability. E{y} and E{y|z} denote, respectively, the expectation of the stochastic variable *y* and the expectation of *y* conditional on *z*. $0_{n\times m}$ represents the n×m zero matrix. $I_{n\times m}$ represents the n×m identity matrix. diag{·} is a block-diagonal matrix. In the symmetric block matrices, “∗” stands for an ellipsis for terms induced by symmetry.

## 2. Problem Formulation and Preliminaries

### 2.1. System Model and Communication Channel

**Figure 1:**
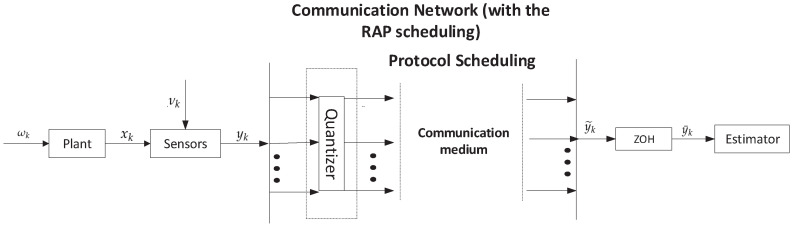
The structure block diagram of the RAP-based filtering scheme.

In this paper, a schematic sketch of the addressed filtering problem for a networked system is depicted in Figure 1 (arrows indicate the signal flow), where the data transmission between the sensors and the remote nonlinear filter is executed via a shared communication channel. It can be seen from Figure 1 that the measurement outputs are transmitted to the remote filter via a communication network. During the network, the signals are first affected by the quantization scheme and then scheduled by the RAP. Finally, the signals enter the remote filter through the zero-order holder (ZOH). In what follows, we will introduce the system model and the communication channel.

Consider the following class of nonlinear stochastic time-delayed systems:

(1) $$\begin{array}{c} \left\{\begin{array}{cc} x_{k+1} & =f\left(x_{k},x_{k-T}\right)+D_{1}\omega_{k}\\ x_{j} & =\varphi_{j},\;j=0,-1,\cdots ,-T\\ y_{k} & =h\left(x_{k}\right)+D_{2}\nu_{k} \end{array}\right. \end{array}$$

where $x_{k}\in R^{n}$ and $y_{k}\in R^{m}$ denote, respectively, the system state and the measurement signal before transmitted via the communication channel; $f\left(\cdot ,\cdot \right):R^{n}\times R^{n}\mapsto R^{n}$ and $h\left(\cdot \right):R^{n}\mapsto R^{m}$ are two nonlinear vector-valued functions; T stands for the known state delay; $\varphi_{j}$ (j=0,−1,⋯,−T) represent the initial conditions; $\nu_{k}\in R^{n_{\nu }}$ and $\omega_{k}\in R^{n_{\omega }}$ denote, respectively, the measurement noise and the process noise; $D_{1}$ and $D_{2}$ are known constant matrices with appropriate dimensions.

**Remark** **1.** *In this paper, it is reasonable to assume that the state time-delay is completely known, since the system dynamics including the information about time-delay can always be obtained by using the mathematical modeling and parameter identification in many practical applications. For the case where only partial information about state time-delay is available (e.g., the bounds of the time-delay), the corresponding filtering problem is always handled by using the robust schemes, see, e.g., [45,46].*

**Assumption** **1.** *The measurement noise $\nu_{k}$ and the process noise $\omega_{k}$, which are mutually uncorrelated, are zero-mean Gaussian white noises with the following statistical properties:*

(2) $$\begin{array}{cc} E\{\omega_{k}\omega_{k}^{T}\}= & \;\bar{L}{\bar{L}}^{T},\;E\left\{\nu_{k}\nu_{k}^{T}\right\}=\bar{\Upsilon }{\bar{\Upsilon }}^{T} \end{array}$$

*where $\bar{L}$ and $\bar{\Upsilon }$ are known time-invariant matrices with proper dimensions.*

**Assumption** **2.** *The functions f(·,·) and h(·) satisfy the conditions: f(0,0)=0, h(0)=0 and*

(3) $$\begin{array}{c} \left\{\begin{array}{cc} \; & ∥f\left(x_{k}+\sigma ,x_{k-T}+\delta \right)-f\left(x_{k},x_{k-T}\right)-\left[\begin{array}{cc} A & B \end{array}\right]\left[\begin{array}{c} \sigma \\ \delta \end{array}\right]∥\leq a_{1}∥\left[\begin{array}{c} \sigma \\ \delta \end{array}\right]∥\\ \; & ∥h\left(x_{k}+\sigma \right)-h\left(x_{k}\right)-C\sigma ∥\leq a_{2}∥\sigma ∥ \end{array}\right. \end{array}$$

*for all vectors $\sigma ,\delta \in R^{n}$, where $A\in R^{n\times n}$, $B\in R^{n\times n}$, and $C\in R^{m\times n}$ are known time-invariant matrices; $a_{1}$ and $a_{2}$ are known positive constants.*

Next, let us discuss the effects incurred by the communication protocol. For technical convenience, the measurement output before being transmitted is rewritten as

(4) $$\begin{array}{c} \begin{array}{c} y_{k}={\left[\begin{array}{cccc} y_{1,k}^{T} & y_{2,k}^{T} & \cdots & y_{m,k}^{T} \end{array}\right]}^{T} \end{array} \end{array}$$

where $y_{j,k}\in R$ (j=1,2,⋯,m) is the measurement output of the *j*th sensor node.

The measurement signal, on the other hand, is firstly quantized by the uniform quantizer, and then sent through the communication channel with the RAP scheduling. Define the quantized measurement signal at time instant *k* as follows:

(5) $$\begin{array}{c} \begin{array}{c} {\tilde{y}}_{k}≜R\left(y_{k}\right)≜{\left[\begin{array}{cccc} {\tilde{y}}_{1,k}^{T} & {\tilde{y}}_{2,k}^{T} & \cdots & {\tilde{y}}_{m,k}^{T} \end{array}\right]}^{T} \end{array} \end{array}$$

where ${\tilde{y}}_{j,k}≜\varpi H\left(\frac{y_{j,k}}{\varpi }\right)\in R$ (j=1,2,⋯,m), ϖ denotes the quantizing level, and H(·) denotes a function that rounds a number to its nearest integer. Letting $\Delta_{k}≜R\left(y_{k}\right)-y_{k}={\tilde{y}}_{k}-y_{k}$, it is not difficult to verify that

(6) $$\begin{array}{c} \begin{array}{c} {∥\Delta_{k}∥}_{\infty }\leq \frac{\varpi }{2}. \end{array} \end{array}$$

**Remark** **2.** *In this paper, we adopt the uniform quantization scheme. The reasons for adopting this scheme are summarized as follows: (1) the uniform quantizer is easier to be realized in the practice due to its relatively simple mechanism; and (2) when the large-amplitude signals are quantized, the signal-to-noise ratio under the uniform quantization scheme is lower than that in the logarithmic quantization case.*

We are now in a position to analyze the effects of RAP scheduling. In this paper, aiming to prevent transmissions from data collisions, only one sensor node is permitted to get access to the communication channel and transmit the measurement signal to the remote filter at each transmission instant. As such, the RAP is employed to orchestrate the order of the data transmissions. Let $\varepsilon_{k}\in \left\{1,2,\cdots ,m\right\}$ represent the current sensor node getting access to the communication channel. Under the effects of the RAP scheduling, $\varepsilon_{k}$ is characterized by a homogeneous Markov chain whose transition probability matrix $\Pi ={\left[\begin{array}{c} p_{ji} \end{array}\right]}_{m\times m}$ is

(7) $$\begin{array}{c} \begin{array}{c} \mathrm{Prob}\left\{\varepsilon_{k+1}=i|\varepsilon_{k}=j\right\}=p_{ji},\;j,i\in \left\{1,2,\cdots ,m\right\} \end{array} \end{array}$$

where $p_{ji}\geq 0$ (j,i∈{1,2,⋯,m}) is the transition probability from node *j* to node *i* and $\sum_{i=1}^{m}p_{ji}=1,\forall j\in \left\{1,2,\cdots ,m\right\}$.

Define the measurement signal after being transmitted as follows:

(8) $$\begin{array}{c} \begin{array}{c} {\bar{y}}_{k}≜{\left[\begin{array}{cccc} {\bar{y}}_{1,k}^{T} & {\bar{y}}_{2,k}^{T} & \cdots & {\bar{y}}_{m,k}^{T} \end{array}\right]}^{T}. \end{array} \end{array}$$

By using the ZOH (a kind of data-holding strategies), the updating rule of ${\bar{y}}_{j,k}$ is described as

(9) $$\begin{array}{c} \begin{array}{c} {\bar{y}}_{j,k}=\left\{\begin{array}{cc} {\tilde{y}}_{j,k},\; & \;j=\varepsilon_{k}\\ {\bar{y}}_{j,k-1}, & \;\mathrm{otherwise}. \end{array}\right. \end{array} \end{array}$$

Accordingly, the measurement signal after transmitted ${\bar{y}}_{k}$ is rewritten as follows:

(10) $$\begin{array}{c} \begin{array}{cc} {\bar{y}}_{k} & =\Phi_{\varepsilon_{k}}{\tilde{y}}_{k}+\left(I-\Phi_{\varepsilon_{k}}\right){\bar{y}}_{k-1} \end{array} \end{array}$$

where $\Phi_{\varepsilon_{k}}≜\mathrm{diag}\left\{\delta \left(\varepsilon_{k}-1\right),\delta \left(\varepsilon_{k}-2\right),\cdots ,\delta \left(\varepsilon_{k}-m\right)\right\}$ and δ(a)∈{0,1} is the Kronecker delta function that equals 1 if a=0 and equals 0 otherwise.

**Remark** **3.** *The RAP is also called the stochastic communication protocol. In general, there are mainly two kinds of stochastic processes to characterize the scheduling behaviors of the RAP, one is the discrete-time Markov chain [3], and the other is the independent and identically distributed sequence of stochastic variables [7]. In this work, the RAP scheduling behaviors are modeled by the discrete-time homogeneous Markov chain.*

### 2.2. Structure of the Filter

In this subsection, we are going to construct a nonlinear filter for the networked system described by (1) under the effects of uniform quantization and RAP scheduling. For convenience, let us denote

(11) $$\begin{array}{c} \begin{array}{cc} \; & \;n\left(k\right)≜\tilde{h}\left(x_{k}\right)-\tilde{h}\left({\hat{x}}_{k}\right),\;\tilde{h}\left(x_{k}\right)≜h\left(x_{k}\right)-Cx_{k},\;\tilde{f}\left(x_{k},x_{k-T}\right)≜f\left(x_{k},x_{k-T}\right)-Ax_{k}-Bx_{k-T},\\ \; & \;l\left(k\right)≜\tilde{f}\left(x_{k},x_{k-T}\right)-\tilde{f}\left({\hat{x}}_{k},{\hat{x}}_{k-T}\right) \end{array} \end{array}$$

where ${\hat{x}}_{k}\in R^{n}$ denotes the estimate of $x_{k}$ which will be clarified later. Then, the nonlinear stochastic time-delayed system (1) can be rewritten as follows:

(12) $$\begin{array}{c} \left\{\begin{array}{cc} x_{k+1}= & \;Ax_{k}+Bx_{k-T}+\tilde{f}\left(x_{k},x_{k-T}\right)+D_{1}\omega_{k}\\ {\bar{y}}_{k}= & \;\Phi_{\varepsilon_{k}}Cx_{k}+\Phi_{\varepsilon_{k}}\tilde{h}\left(x_{k}\right)+\Phi_{\varepsilon_{k}}D_{2}\nu_{k}+\Phi_{\varepsilon_{k}}\Delta_{k}+\left(I-\Phi_{\varepsilon_{k}}\right){\bar{y}}_{k-1}. \end{array}\right. \end{array}$$

Letting ${\bar{x}}_{k+1}≜{\left[\begin{array}{cc} x_{k+1}^{T} & {\bar{y}}_{k}^{T} \end{array}\right]}^{T}$, we have

(13) $$\begin{array}{c} \left\{\begin{array}{cc} {\bar{x}}_{k+1}= & \;{\tilde{A}}_{\varepsilon_{k}}{\bar{x}}_{k}+{\tilde{B}}_{\varepsilon_{k}}\tilde{f}\left({\bar{x}}_{k}\right)+{\tilde{C}}_{\varepsilon_{k}}{\tilde{\omega }}_{k}+{\tilde{D}}_{\varepsilon_{k}}{\bar{x}}_{k-T}\\ {\bar{y}}_{k}= & \;{\bar{A}}_{\varepsilon_{k}}{\bar{x}}_{k}+{\bar{B}}_{\varepsilon_{k}}\tilde{f}\left({\bar{x}}_{k}\right)+{\bar{C}}_{\varepsilon_{k}}{\tilde{\omega }}_{k} \end{array}\right. \end{array}$$

where

$$\begin{array}{cc} \; & \tilde{f}\left({\bar{x}}_{k}\right)≜{\left[\begin{array}{cc} {\tilde{f}}^{T}\left(x_{k},x_{k-T}\right) & {\tilde{h}}^{T}\left(x_{k}\right) \end{array}\right]}^{T},\;{\tilde{\omega }}_{k}≜{\left[\begin{array}{ccc} \omega_{k}^{T} & \nu_{k}^{T} & \Delta_{k}^{T} \end{array}\right]}^{T},\\ \; & {\tilde{A}}_{\varepsilon_{k}}≜\left[\begin{array}{cc} A & 0_{n\times m}\\ \Phi_{\varepsilon_{k}}C & I_{m\times m}-\Phi_{\varepsilon_{k}} \end{array}\right],\;{\tilde{B}}_{\varepsilon_{k}}≜\left[\begin{array}{cc} I_{n\times n} & 0_{n\times m}\\ 0_{m\times n} & \Phi_{\varepsilon_{k}} \end{array}\right],\;{\bar{A}}_{\varepsilon_{k}}≜\left[\begin{array}{cc} \Phi_{\varepsilon_{k}}C & I_{m\times m}-\Phi_{\varepsilon_{k}} \end{array}\right],\\ \; & {\bar{B}}_{\varepsilon_{k}}≜\left[\begin{array}{cc} 0_{m\times n} & \Phi_{\varepsilon_{k}} \end{array}\right],\;{\tilde{C}}_{\varepsilon_{k}}≜\left[\begin{array}{ccc} D_{1} & 0_{n\times n_{\nu }} & 0_{n\times m}\\ 0_{m\times n_{\omega }} & \Phi_{\varepsilon_{k}}D_{2} & \Phi_{\varepsilon_{k}} \end{array}\right],\;{\tilde{D}}_{\varepsilon_{k}}≜\left[\begin{array}{cc} B & 0_{n\times m}\\ 0_{m\times n} & 0_{m\times m} \end{array}\right],\\ \; & {\bar{C}}_{\varepsilon_{k}}≜\left[\begin{array}{ccc} 0_{m\times n_{\omega }} & \Phi_{\varepsilon_{k}}D_{2} & \Phi_{\varepsilon_{k}} \end{array}\right]. \end{array}$$

In this paper, the filter is constructed for the augmented system (13) as the following form

(14) $$\begin{array}{c} \left\{\begin{array}{cc} {\hat{\bar{x}}}_{k+1}= & \;{\tilde{A}}_{\varepsilon_{k}}{\hat{\bar{x}}}_{k}+{\tilde{B}}_{\varepsilon_{k}}\tilde{f}\left({\hat{\bar{x}}}_{k}\right)+{\tilde{D}}_{\varepsilon_{k}}{\hat{\bar{x}}}_{k-T}+K_{\varepsilon_{k}}\left({\bar{y}}_{k}-{\hat{y}}_{k}\right)\\ {\hat{y}}_{k}= & \;{\bar{A}}_{\varepsilon_{k}}{\hat{\bar{x}}}_{k}+{\bar{B}}_{\varepsilon_{k}}\tilde{f}\left({\hat{\bar{x}}}_{k}\right) \end{array}\right. \end{array}$$

where ${\hat{\bar{x}}}_{k}≜{\left[\begin{array}{cc} {\hat{x}}_{k}^{T} & {\hat{y}}_{k-1}^{T} \end{array}\right]}^{T}$ is the estimate of ${\bar{x}}_{k}$ and $K_{\varepsilon_{k}}\in R^{(m+n)\times m}$ is the filter gain matrix to be designed.

**Remark** **4.** *In this paper, a switch approach is adopted to deal with the effects caused by the RAP scheduling. By taking fully the dynamical behavior of the RAP into account, we design a switch-signal-dependent piecewise filter to achieve the prescribed filtering task. As the real plant runs, the side of filter can detect that which sensor node is connected to the communication network, and then activates the related filter. Such a kind of filter posses more flexibility and is easy to be implemented in the practice.*

Letting $e_{k}≜{\bar{x}}_{k}-{\hat{\bar{x}}}_{k}$, the filtering error dynamics is given by

(15) $$\begin{array}{c} \begin{array}{cc} e_{k+1}= & \;{\bar{x}}_{k+1}-{\hat{\bar{x}}}_{k+1}\\ = & \;{\tilde{A}}_{\varepsilon_{k}}{\bar{x}}_{k}+{\tilde{B}}_{\varepsilon_{k}}\tilde{f}\left({\bar{x}}_{k}\right)+{\tilde{C}}_{\varepsilon_{k}}{\tilde{\omega }}_{k}+{\tilde{D}}_{\varepsilon_{k}}{\bar{x}}_{k-T}-{\tilde{A}}_{\varepsilon_{k}}{\hat{\bar{x}}}_{k}-{\tilde{B}}_{\varepsilon_{k}}\tilde{f}\left({\hat{\bar{x}}}_{k}\right)-{\tilde{D}}_{\varepsilon_{k}}{\hat{\bar{x}}}_{k-T}-K_{\varepsilon_{k}}\left({\bar{y}}_{k}-{\hat{y}}_{k}\right)\\ = & \;{\tilde{A}}_{\varepsilon_{k}}{\bar{x}}_{k}+{\tilde{B}}_{\varepsilon_{k}}\tilde{f}\left({\bar{x}}_{k}\right)+{\tilde{C}}_{\varepsilon_{k}}{\tilde{\omega }}_{k}+{\tilde{D}}_{\varepsilon_{k}}{\bar{x}}_{k-T}-{\tilde{A}}_{\varepsilon_{k}}{\hat{\bar{x}}}_{k}-{\tilde{B}}_{\varepsilon_{k}}\tilde{f}\left({\hat{\bar{x}}}_{k}\right)-{\tilde{D}}_{\varepsilon_{k}}{\hat{\bar{x}}}_{k-T}\\ \; & \;-K_{\varepsilon_{k}}\left({\bar{A}}_{\varepsilon_{k}}{\bar{x}}_{k}+{\bar{B}}_{\varepsilon_{k}}\tilde{f}\left({\bar{x}}_{k}\right)+{\bar{C}}_{\varepsilon_{k}}{\tilde{\omega }}_{k}-{\bar{A}}_{\varepsilon_{k}}{\hat{\bar{x}}}_{k}-{\bar{B}}_{\varepsilon_{k}}\tilde{f}\left({\hat{\bar{x}}}_{k}\right)\right)\\ = & \;{\tilde{A}}_{\varepsilon_{k}}e_{k}+{\tilde{B}}_{\varepsilon_{k}}\tilde{f}\left(e_{k}\right)+{\tilde{C}}_{\varepsilon_{k}}{\tilde{\omega }}_{k}+{\tilde{D}}_{\varepsilon_{k}}e_{k-T}-K_{\varepsilon_{k}}\left({\bar{A}}_{\varepsilon_{k}}e_{k}+{\bar{B}}_{\varepsilon_{k}}\tilde{f}\left(e_{k}\right)+{\bar{C}}_{\varepsilon_{k}}{\tilde{\omega }}_{k}\right)\\ = & \;{\vec{A}}_{\varepsilon_{k}}e_{k}+{\vec{B}}_{\varepsilon_{k}}\tilde{f}\left(e_{k}\right)+{\vec{C}}_{\varepsilon_{k}}{\tilde{\omega }}_{k}+{\vec{D}}_{\varepsilon_{k}}e_{k-T} \end{array} \end{array}$$

where

$$\begin{array}{cc} \; & \tilde{f}\left(e_{k}\right)≜{\left[\begin{array}{cc} l_{k}^{T} & n_{k}^{T} \end{array}\right]}^{T},\;{\vec{A}}_{\varepsilon_{k}}≜{\tilde{A}}_{\varepsilon_{k}}-K_{\varepsilon_{k}}{\bar{A}}_{\varepsilon_{k}},\;{\vec{B}}_{\varepsilon_{k}}≜{\tilde{B}}_{\varepsilon_{k}}-K_{\varepsilon_{k}}{\bar{B}}_{\varepsilon_{k}},\\ \; & {\vec{C}}_{\varepsilon_{k}}≜{\tilde{C}}_{\varepsilon_{k}}-K_{\varepsilon_{k}}{\bar{C}}_{\varepsilon_{k}},\;\;{\vec{D}}_{\varepsilon_{k}}≜{\tilde{D}}_{\varepsilon_{k}}. \end{array}$$

Before proceeding further, we introduce the following definition concerning the exponential ultimate boundedness (EUB).

**Definition** **1.** *Let $e_{k;\iota }$ represent the state trajectory of the filtering error system (15) from the initial data $e_{\theta }≜\iota_{\theta }$(−T≤θ≤0). The filtering error is said to be EUBMS if there exist constants η>0, β∈[0,1), κ≥0 such that*

(16) $$\begin{array}{c} \begin{array}{cc} E\{∥e_{k;\iota }{∥}^{2}\}\leq & \;\eta \beta^{k}\underset{-T\leq \theta \leq 0}{\sup}E\{∥\iota_{\theta }{∥}^{2}\}+\kappa \end{array} \end{array}$$

*where κ is an asymptotic upper bound.*

The objective of this paper is to devise a filter of the form (15) to estimate the state of the system (13) such that the dynamics of the filtering error is EUBMS.

## 3. Main Results

In this section, a sufficient condition is established to guarantee the EUB of the system (15) in mean square. Moreover, the desired filter parameters are obtained by solving a set of LMIs.

Now, we are in a position to consider the EUB of the system (15).

**Theorem** **1.** *Let the filter gain matrices $K_{j}$(j=1,2,⋯,m) be given. Assume that there exist m+3 positive definite matrices $P_{j}\in R^{\left(n+m)\times (n+m\right)}$(j=1,2,⋯,m), $Q\in R^{\left(n+m)\times (n+m\right)}$, $H\in R^{n_{\omega }\times n_{\omega }}$, $T\in R^{n_{\nu }\times n_{\nu }}$, four positive scalars $\gamma_{\varsigma }$(ς=1,2,3), α≤1, such that the following matrix inequalities hold, for j=1,2,⋯,m:*

(17) $$\begin{array}{c} \Lambda^{j}=\left[\begin{array}{ccccc} \Lambda_{11}^{j} & \Lambda_{12}^{j} & \Lambda_{13}^{j} & \Lambda_{14}^{j} & 0\\ {}* & \Lambda_{22}^{j} & \Lambda_{23}^{j} & \Lambda_{24}^{j} & 0\\ {}* & * & \Lambda_{33}^{j} & \Lambda_{34}^{j} & 0\\ {}* & * & * & \Lambda_{44}^{j} & 0\\ {}* & * & * & * & \Lambda_{55}^{j} \end{array}\right]<0 \end{array}$$

*where*

$$\begin{array}{cc} A_{331}^{j}≜ & \;\left[\begin{array}{ccc} I_{n_{\omega }\times n_{\omega }} & 0_{n_{\omega }\times n_{\nu }} & 0_{n_{\omega }\times m} \end{array}\right],\;\;A_{332}^{j}≜\left[\begin{array}{ccc} 0_{n_{\nu }\times n_{\omega }} & I_{n_{\nu }\times n_{\nu }} & 0_{n_{\nu }\times m} \end{array}\right],\;\;\;\;A_{112}^{j}≜\left[\begin{array}{cc} a_{2}^{2}I_{n\times n} & 0_{n\times m}\\ 0_{m\times n} & 0_{m\times m} \end{array}\right],\\ A_{221}^{j}≜ & \;\left[\begin{array}{cc} I_{n\times n} & 0_{n\times m}\\ 0_{m\times n} & 0_{m\times m} \end{array}\right],\;\;A_{111}^{j}≜\left[\begin{array}{cc} a_{1}^{2}I_{n\times n} & 0_{n\times m}\\ 0_{m\times n} & 0_{m\times m} \end{array}\right],\;\;A_{222}^{j}≜\left[\begin{array}{cc} 0_{n\times n} & 0_{n\times m}\\ 0_{m\times n} & I_{m\times m} \end{array}\right],\\ A_{333}^{j}≜ & \;\left[\begin{array}{ccc} 0_{m\times n_{\omega }} & 0_{m\times n_{\nu }} & I_{m\times m} \end{array}\right],\;\;A_{441}^{j}≜\left[\begin{array}{cc} a_{1}^{2}I_{n\times n} & 0_{n\times m}\\ 0_{m\times n} & 0_{m\times m} \end{array}\right],\\ \Lambda_{13}^{j}≜ & \;{{\vec{A}}_{j}}^{T}\sum_{i=1}^{m}p_{ji}P_{i}{\vec{C}}_{j},\;\;\Lambda_{11}^{j}≜{{\vec{A}}_{j}}^{T}\sum_{i=1}^{m}p_{ji}P_{i}{\vec{A}}_{j}+TQ-P_{j}+\gamma_{1}A_{111}^{j}+\gamma_{2}A_{112}^{j}+\alpha P_{j},\\ \Lambda_{12}^{j}≜ & \;{{\vec{A}}_{j}}^{T}\sum_{i=1}^{m}p_{ji}P_{i}{\vec{B}}_{j},\;\;\Lambda_{14}^{j}≜{{\vec{A}}_{j}}^{T}\sum_{i=1}^{m}p_{ji}P_{i}{\vec{D}}_{j},\;\;\Lambda_{34}^{j}≜{{\vec{C}}_{j}}^{T}\sum_{i=1}^{m}p_{ji}P_{i}{\vec{D}}_{j},\\ \Lambda_{22}^{j}≜ & \;{{\vec{B}}_{j}}^{T}\sum_{i=1}^{m}p_{ji}P_{i}{\vec{B}}_{j}-\gamma_{1}A_{221}^{j}-\gamma_{2}A_{222}^{j},\;\;\Lambda_{23}^{j}≜{{\vec{B}}_{j}}^{T}\sum_{i=1}^{m}p_{ji}P_{i}{\vec{C}}_{j},\;\;\Lambda_{24}^{j}≜{{\vec{B}}_{j}}^{T}\sum_{i=1}^{m}p_{ji}P_{i}{\vec{D}}_{j},\\ \Lambda_{44}^{j}≜ & \;{{\vec{D}}_{j}}^{T}\sum_{i=1}^{m}p_{ji}P_{i}{\vec{D}}_{j}-Q+\gamma_{1}A_{441}^{j}+\alpha Q,\;\;\Lambda_{55}^{j}≜\mathrm{diag}\left\{-Q+2\alpha Q,-Q+3\alpha Q,\cdots ,-Q+T\alpha Q\right\},\\ \Lambda_{33}^{j}≜ & \;{{\vec{C}}_{j}}^{T}\sum_{i=1}^{m}p_{ji}P_{i}{\vec{C}}_{j}-{A_{331}^{j}}^{T}HA_{331}^{j}-{A_{332}^{j}}^{T}TA_{332}^{j}-{A_{333}^{j}}^{T}\gamma_{3}A_{333}^{j}. \end{array}$$

Then, the filtering error dynamics (15) is EUBMS.

**Proof** **of** **Theorem** **1.** In order to analyze the EUB of the system (15), we choose the Lyapunov-like functional as follows:

(18) $$\begin{array}{c} V_{k}=e_{k}^{T}P_{\varepsilon_{k}}e_{k}+\sum_{\varrho =0}^{T-1}\sum_{j=k-T+\varrho }^{k-1}e_{j}^{T}Qe_{j}. \end{array}$$

Then, we have

(19) $$\begin{array}{cc} V_{k+1}= & \;e_{k+1}^{T}P_{\varepsilon_{k+1}}e_{k+1}+\sum_{\varrho =0}^{T-1}\sum_{j=k-T+\varrho +1}^{k}e_{j}^{T}Qe_{j}. \end{array}$$

Letting $\varepsilon_{k}=j$, it follows from (18) and (19) that

(20) $$\begin{array}{c} \begin{array}{cc} \Delta V_{k}= & \;V_{k+1}-V_{k}\\ = & \;e_{k+1}^{T}P_{\varepsilon_{k+1}}e_{k+1}+\sum_{\varrho =0}^{T-1}\sum_{j=k-T+\varrho +1}^{k}e_{j}^{T}Qe_{j}-e_{k}^{T}P_{\varepsilon_{k}}e_{k}-\sum_{\varrho =0}^{T-1}\sum_{j=k-T+\varrho }^{k-1}e_{j}^{T}Qe_{j}\\ = & \;e_{k+1}^{T}P_{\varepsilon_{k+1}}e_{k+1}-e_{k}^{T}P_{\varepsilon_{k}}e_{k}+\sum_{\varrho =0}^{T-1}\sum_{j=k-T+\varrho +1}^{k}e_{j}^{T}Qe_{j}-\sum_{\varrho =0}^{T-1}\sum_{j=k-T+\varrho }^{k-1}e_{j}^{T}Qe_{j}\\ = & \;e_{k+1}^{T}P_{\varepsilon_{k+1}}e_{k+1}-e_{k}^{T}P_{\varepsilon_{k}}e_{k}+e_{k}^{T}Qe_{k}-e_{k-T}^{T}Qe_{k-T}+e_{k}^{T}Qe_{k}-e_{k-T+1}^{T}Qe_{k-T+1}\\ \; & \;+\cdots +e_{k}^{T}Qe_{k}-e_{k-2}^{T}Qe_{k-2}+e_{k}^{T}Qe_{k}-e_{k-1}^{T}Qe_{k-1}\\ = & \;e_{k+1}^{T}P_{\varepsilon_{k+1}}e_{k+1}+Te_{k}^{T}Qe_{k}-e_{k}^{T}P_{\varepsilon_{k}}e_{k}-e_{k-T}^{T}Qe_{k-T}-e_{k-T+1}^{T}Qe_{k-T+1}\\ \; & \;-\cdots -e_{k-2}^{T}Qe_{k-2}-e_{k-1}^{T}Qe_{k-1}\\ = & \;\left(e_{k}^{T}{{\vec{A}}_{\varepsilon_{k}}}^{T}+{\tilde{f}}^{T}\left(e_{k}\right){{\vec{B}}_{\varepsilon_{k}}}^{T}+{\tilde{\omega }}_{k}^{T}{{\vec{C}}_{\varepsilon_{k}}}^{T}+e_{k-T}^{T}{{\vec{D}}_{\varepsilon_{k}}}^{T}\right)P_{\varepsilon_{k+1}}\left({\vec{A}}_{\varepsilon_{k}}e_{k}+{\vec{B}}_{\varepsilon_{k}}\tilde{f}\left(e_{k}\right)+{\vec{C}}_{\varepsilon_{k}}{\tilde{\omega }}_{k}+{\vec{D}}_{\varepsilon_{k}}e_{k-T}\right)\\ \; & \;+Te_{k}^{T}Qe_{k}-e_{k}^{T}P_{\varepsilon_{k}}e_{k}-e_{k-T}^{T}Qe_{k-T}-e_{k-T+1}^{T}Qe_{k-T+1}-\cdots -e_{k-1}^{T}Qe_{k-1}\\ = & \;e_{k}^{T}{{\vec{A}}_{\varepsilon_{k}}}^{T}P_{\varepsilon_{k+1}}{\vec{A}}_{\varepsilon_{k}}e_{k}+e_{k}^{T}{{\vec{A}}_{\varepsilon_{k}}}^{T}P_{\varepsilon_{k+1}}{\vec{B}}_{\varepsilon_{k}}\tilde{f}\left(e_{k}\right)+e_{k}^{T}{{\vec{A}}_{\varepsilon_{k}}}^{T}P_{\varepsilon_{k+1}}{\vec{C}}_{\varepsilon_{k}}{\tilde{\omega }}_{k}+e_{k}^{T}{{\vec{A}}_{\varepsilon_{k}}}^{T}P_{\varepsilon_{k+1}}{\vec{D}}_{\varepsilon_{k}}e_{k-T}\\ \; & \;+{\tilde{f}}^{T}\left(e_{k}\right){{\vec{B}}_{\varepsilon_{k}}}^{T}P_{\varepsilon_{k+1}}{\vec{A}}_{\varepsilon_{k}}e_{k}+{\tilde{f}}^{T}\left(e_{k}\right){{\vec{B}}_{\varepsilon_{k}}}^{T}P_{\varepsilon_{k+1}}{\vec{B}}_{\varepsilon_{k}}\tilde{f}\left(e_{k}\right)+{\tilde{f}}^{T}\left(e_{k}\right){{\vec{B}}_{\varepsilon_{k}}}^{T}P_{\varepsilon_{k+1}}{\vec{C}}_{\varepsilon_{k}}{\tilde{\omega }}_{k}\\ \; & \;+{\tilde{f}}^{T}\left(e_{k}\right){{\vec{B}}_{\varepsilon_{k}}}^{T}P_{\varepsilon_{k+1}}{\vec{D}}_{\varepsilon_{k}}e_{k-T}+{\tilde{\omega }}_{k}^{T}{{\vec{C}}_{\varepsilon_{k}}}^{T}P_{\varepsilon_{k+1}}{\vec{A}}_{\varepsilon_{k}}e_{k}+{\tilde{\omega }}_{k}^{T}{{\vec{C}}_{\varepsilon_{k}}}^{T}P_{\varepsilon_{k+1}}{\vec{B}}_{\varepsilon_{k}}\tilde{f}\left(e_{k}\right)+{\tilde{\omega }}_{k}^{T}{{\vec{C}}_{\varepsilon_{k}}}^{T}P_{\varepsilon_{k+1}}{\vec{C}}_{\varepsilon_{k}}{\tilde{\omega }}_{k}\\ \; & \;+{\tilde{\omega }}_{k}^{T}{{\vec{C}}_{\varepsilon_{k}}}^{T}P_{\varepsilon_{k+1}}{\vec{D}}_{\varepsilon_{k}}e_{k-T}+e_{k-T}^{T}{{\vec{D}}_{\varepsilon_{k}}}^{T}P_{\varepsilon_{k+1}}{\vec{A}}_{\varepsilon_{k}}e_{k}+e_{k-T}^{T}{{\vec{D}}_{\varepsilon_{k}}}^{T}P_{\varepsilon_{k+1}}{\vec{B}}_{\varepsilon_{k}}\tilde{f}\left(e_{k}\right)+e_{k-T}^{T}{{\vec{D}}_{\varepsilon_{k}}}^{T}P_{\varepsilon_{k+1}}{\vec{C}}_{\varepsilon_{k}}{\tilde{\omega }}_{k}\\ \; & \;+e_{k-T}^{T}{{\vec{D}}_{\varepsilon_{k}}}^{T}P_{\varepsilon_{k+1}}{\vec{D}}_{\varepsilon_{k}}e_{k-T}+Te_{k}^{T}Qe_{k}-e_{k}^{T}P_{\varepsilon_{k}}e_{k}-e_{k-T}^{T}Qe_{k-T}-e_{k-T+1}^{T}Qe_{k-T+1}\\ \; & \;-\cdots -e_{k-1}^{T}Qe_{k-1}\\ = & \;\xi_{k}^{T}\left[\begin{array}{ccccc} h_{j}^{11} & h_{j}^{12} & h_{j}^{13} & h_{j}^{14} & 0\\ {}* & h_{j}^{22} & h_{j}^{23} & h_{j}^{24} & 0\\ {}* & * & h_{j}^{33} & h_{j}^{34} & 0\\ {}* & * & * & h_{j}^{44} & 0\\ {}* & * & * & * & h_{j}^{55} \end{array}\right]\xi_{k}\\ = & \;\xi_{k}^{T}{\left[h_{j}^{pq}\right]}_{5\times 5}\xi_{k} \end{array} \end{array}$$

where

$$\begin{array}{cc} \xi_{k} & ≜\left[\begin{array}{c} e_{k}\\ \tilde{f}\left(e_{k}\right)\\ {\tilde{\omega }}_{k}\\ e_{k-T}\\ {\overset{´}{e}}_{k-1} \end{array}\right],\;h_{j}^{11}≜{{\vec{A}}_{j}}^{T}\sum_{i=1}^{m}p_{ji}P_{i}{\vec{A}}_{j}+TQ-P_{j},\;{\overset{´}{e}}_{k-1}≜\left[\begin{array}{c} e_{k-T+1}^{T}\\ e_{k-T+2}^{T}\\ \vdots \\ e_{k-1}^{T} \end{array}\right],\;\\ h_{j}^{12} & ≜{{\vec{A}}_{j}}^{T}\sum_{i=1}^{m}p_{ji}P_{i}{\vec{B}}_{j},\;h_{j}^{13}≜{{\vec{A}}_{j}}^{T}\sum_{i=1}^{m}p_{ji}P_{i}{\vec{C}}_{j},\;h_{j}^{14}≜{{\vec{A}}_{j}}^{T}\sum_{i=1}^{m}p_{ji}P_{i}{\vec{D}}_{j},\;h_{j}^{22}≜{{\vec{B}}_{j}}^{T}\sum_{i=1}^{m}p_{ji}P_{i}{\vec{B}}_{j},\\ h_{j}^{23} & ≜{{\vec{B}}_{j}}^{T}\sum_{i=1}^{m}p_{ji}P_{i}{\vec{C}}_{j},\;h_{j}^{24}≜{{\vec{B}}_{j}}^{T}\sum_{i=1}^{m}p_{ji}P_{i}{\vec{D}}_{j},\;h_{j}^{33}≜{{\vec{C}}_{j}}^{T}\sum_{i=1}^{m}p_{ji}P_{i}{\vec{C}}_{j},\;h_{j}^{34}≜{{\vec{C}}_{j}}^{T}\sum_{i=1}^{m}p_{ji}P_{i}{\vec{D}}_{j},\\ h_{j}^{44} & ≜{{\vec{D}}_{j}}^{T}\sum_{i=1}^{m}p_{ji}P_{i}{\vec{D}}_{j}-Q,\;h_{j}^{55}≜{\mathrm{diag}}_{T-1}\left\{-Q\right\}. \end{array}$$

Furthermore, one can infer from (3) and (11) that

(21) $$\begin{array}{cc} \; & \gamma_{1}{\left(f\left(x_{k},x_{k-T}\right)-f\left({\hat{x}}_{k},{\hat{x}}_{k-T}\right)-\left[\begin{array}{cc} A & B \end{array}\right]\left[\begin{array}{c} x_{k}-{\hat{x}}_{k}\\ x_{k-T}-{\hat{x}}_{k-T} \end{array}\right]\right)}^{T}(f\left(x_{k},x_{k-T}\right)\\ \; & \;-f\left({\hat{x}}_{k},{\hat{x}}_{k-T}\right)-\left[\begin{array}{cc} A & B \end{array}\right]\left[\begin{array}{c} x_{k}-{\hat{x}}_{k}\\ x_{k-T}-{\hat{x}}_{k-T} \end{array}\right])\\ = & \gamma_{1}{\left(\tilde{f}\left(x_{k},x_{k-T}\right)+Ax_{k}+Bx_{k-T}-\tilde{f}\left({\hat{x}}_{k},{\hat{x}}_{k-T}\right)-A{\hat{x}}_{k}-B{\hat{x}}_{k-T}-\left[\begin{array}{cc} A & B \end{array}\right]\left[\begin{array}{c} x_{k}-{\hat{x}}_{k}\\ x_{k-T}-{\hat{x}}_{k-T} \end{array}\right]\right)}^{T}\\ \; & \times \left(\tilde{f}\left(x_{k},x_{k-T}\right)+Ax_{k}+Bx_{k-T}-\tilde{f}\left({\hat{x}}_{k},{\hat{x}}_{k-T}\right)-A{\hat{x}}_{k}-B{\hat{x}}_{k-T}-\left[\begin{array}{cc} A & B \end{array}\right]\left[\begin{array}{c} x_{k}-{\hat{x}}_{k}\\ x_{k-T}-{\hat{x}}_{k-T} \end{array}\right]\right)\\ = & \gamma_{1}{\left(\tilde{f}\left(x_{k},x_{k-T}\right)-\tilde{f}\left({\hat{x}}_{k},{\hat{x}}_{k-T}\right)+A\left(x_{k}-{\hat{x}}_{k}\right)+B\left(x_{k-T}-{\hat{x}}_{k-T}\right)-\left[\begin{array}{cc} A & B \end{array}\right]\left[\begin{array}{c} x_{k}-{\hat{x}}_{k}\\ x_{k-T}-{\hat{x}}_{k-T} \end{array}\right]\right)}^{T}\\ \; & \times \left(\tilde{f}\left(x_{k},x_{k-T}\right)-\tilde{f}\left({\hat{x}}_{k},{\hat{x}}_{k-T}\right)+A\left(x_{k}-{\hat{x}}_{k}\right)+B\left(x_{k-T}-{\hat{x}}_{k-T}\right)-\left[\begin{array}{cc} A & B \end{array}\right]\left[\begin{array}{c} x_{k}-{\hat{x}}_{k}\\ x_{k-T}-{\hat{x}}_{k-T} \end{array}\right]\right)\\ = & \gamma_{1}{\left(l_{k}+A\left(x_{k}-{\hat{x}}_{k}\right)+B\left(x_{k-T}-{\hat{x}}_{k-T}\right)-\left[\begin{array}{cc} A & B \end{array}\right]\left[\begin{array}{c} x_{k}-{\hat{x}}_{k}\\ x_{k-T}-{\hat{x}}_{k-T} \end{array}\right]\right)}^{T}\\ \; & \times \left(l_{k}+A\left(x_{k}-{\hat{x}}_{k}\right)+B\left(x_{k-T}-{\hat{x}}_{k-T}\right)-\left[\begin{array}{cc} A & B \end{array}\right]\left[\begin{array}{c} x_{k}-{\hat{x}}_{k}\\ x_{k-T}-{\hat{x}}_{k-T} \end{array}\right]\right)\\ = & \gamma_{1}{\left(l_{k}+A\left(x_{k}-{\hat{x}}_{k}\right)+B\left(x_{k-T}-{\hat{x}}_{k-T}\right)-A\left(x_{k}-{\hat{x}}_{k}\right)-B\left(x_{k-T}-{\hat{x}}_{k-T}\right)\right)}^{T}\\ \; & \times \left(l_{k}+A\left(x_{k}-{\hat{x}}_{k}\right)+B\left(x_{k-T}-{\hat{x}}_{k-T}\right)-A\left(x_{k}-{\hat{x}}_{k}\right)-B\left(x_{k-T}-{\hat{x}}_{k-T}\right)\right)\\ = & \gamma_{1}l_{k}^{T}\;l_{k}\\ = & \gamma_{1}{\tilde{f}}^{T}\left(e_{k}\right)A_{221}^{j}\tilde{f}\left(e_{k}\right)\\ \leq & \gamma_{1}a_{1}^{2}{\left[\begin{array}{c} x_{k}-{\hat{x}}_{k}\\ x_{k-T}-{\hat{x}}_{k-T} \end{array}\right]}^{T}\left[\begin{array}{c} x_{k}-{\hat{x}}_{k}\\ x_{k-T}-{\hat{x}}_{k-T} \end{array}\right]\\ = & \gamma_{1}a_{1}^{2}{\left(x_{k-T}-{\hat{x}}_{k-T}\right)}^{T}\left(x_{k-T}-{\hat{x}}_{k-T}\right)+\gamma_{1}a_{1}^{2}{\left(x_{k}-{\hat{x}}_{k}\right)}^{T}\left(x_{k}-{\hat{x}}_{k}\right)\\ = & \gamma_{1}e_{k}^{T}A_{111}^{j}e_{k}+\gamma_{1}e_{k-T}^{T}A_{441}^{j}e_{k-T} \end{array}$$

and

(22) $$\begin{array}{c} \begin{array}{cc} \; & \gamma_{2}{\left(h\left(x_{k}\right)-h\left({\hat{x}}_{k}\right)-C\left(x_{k}-{\hat{x}}_{k}\right)\right)}^{T}\left(h\left(x_{k}\right)-h\left({\hat{x}}_{k}\right)-C\left(x_{k}-{\hat{x}}_{k}\right)\right)\\ = & {\gamma_{2}\left(\tilde{h}\left(x_{k}\right)+Cx_{k}-\tilde{h}\left({\hat{x}}_{k}\right)-C{\hat{x}}_{k}-C\left(x_{k}-{\hat{x}}_{k}\right)\right)}^{T}\left(\tilde{h}\left(x_{k}\right)+Cx_{k}-\tilde{h}\left({\hat{x}}_{k}\right)-C{\hat{x}}_{k}-C\left(x_{k}-{\hat{x}}_{k}\right)\right)\\ = & {\gamma_{2}\left(\tilde{h}\left(x_{k}\right)-\tilde{h}\left({\hat{x}}_{k}\right)+Cx_{k}-C{\hat{x}}_{k}-C\left(x_{k}-{\hat{x}}_{k}\right)\right)}^{T}\left(\tilde{h}\left(x_{k}\right)-\tilde{h}\left({\hat{x}}_{k}\right)+Cx_{k}-C{\hat{x}}_{k}-C\left(x_{k}-{\hat{x}}_{k}\right)\right)\\ = & \gamma_{2}{\left(n_{k}+C\left(x_{k}-{\hat{x}}_{k}\right)-C\left(x_{k}-{\hat{x}}_{k}\right)\right)}^{T}\left(n_{k}+C\left(x_{k}-{\hat{x}}_{k}\right)-C\left(x_{k}-{\hat{x}}_{k}\right)\right)\\ = & \gamma_{2}n_{k}^{T}n_{k}\\ = & \gamma_{2}{\tilde{f}}^{T}\left(e_{k}\right)A_{222}^{j}\tilde{f}\left(e_{k}\right)\\ \leq & \gamma_{2}a_{2}^{2}{\left(x_{k}-{\hat{x}}_{k}\right)}^{T}\left(x_{k}-{\hat{x}}_{k}\right)\\ = & \gamma_{2}e_{k}^{T}A_{112}^{j}e_{k}. \end{array} \end{array}$$

Therefore, we have

(23) $$\begin{array}{cc} \Delta V_{k}= & \;\xi_{k}^{T}{\left[h_{j}^{pq}\right]}_{5\times 5}\xi_{k}-\alpha V_{k}+\gamma_{3}{∥\Delta_{k}∥}^{2}+\omega_{k}^{T}H\omega_{k}+\nu_{k}^{T}T\nu_{k}+\alpha V_{k}\\ \; & \;-\gamma_{3}{∥\Delta_{k}∥}^{2}-\omega_{k}^{T}H\omega_{k}-\nu_{k}^{T}T\nu_{k}\\ = & \;(\xi_{k}^{T}{\left[h_{j}^{pq}\right]}_{5\times 5}\xi_{k}+\alpha V_{k}-\gamma_{3}∥\Delta_{k}{∥}^{2}-\omega_{k}^{T}H\omega_{k}-\nu_{k}^{T}T\nu_{k})-\alpha V_{k}\\ \; & \;+\gamma_{3}{∥\Delta_{k}∥}^{2}+\omega_{k}^{T}H\omega_{k}+\nu_{k}^{T}T\nu_{k}\\ = & \;\xi_{k}^{T}\Lambda^{j}\xi_{k}-\alpha V_{k}+\gamma_{3}{∥\Delta_{k}∥}^{2}+\omega_{k}^{T}H\omega_{k}+\nu_{k}^{T}T\nu_{k}\\ \leq & \;-\alpha V_{k}+\gamma_{3}{∥\Delta_{k}∥}^{2}+\omega_{k}^{T}H\omega_{k}+\nu_{k}^{T}T\nu_{k}, \end{array}$$

which indicates that

(24) $$\begin{array}{c} \begin{array}{c} E\left\{\Delta V_{k}|\varepsilon_{k}=j\right\}\leq -\alpha E\left\{V_{k}|\varepsilon_{k}=j\right\}+\zeta \end{array} \end{array}$$

where $\zeta =\gamma_{3}\frac{\varpi^{2}}{4}+\mathrm{tr}\left\{{\bar{L}}^{T}H\bar{L}+{\bar{\Upsilon }}^{T}T\bar{\Upsilon }\right\}$. Furthermore, for any scalar μ≥0, it can be obtained that

(25) $$\begin{array}{c} \begin{array}{cc} \; & \;E\{\mu^{k+1}\Delta V_{k}|\varepsilon_{k}=j\}\\ = & \;E\{\mu^{k+1}V_{k+1}-\mu^{k+1}V_{k}|\varepsilon_{k}=j\}\\ = & \;\mu^{k+1}\left(E\left\{V_{k+1}|\varepsilon_{k}=j\right\}-E\left\{V_{k}|\varepsilon_{k}=j\right\}\right)+\mu^{k}E\left\{V_{k}|\varepsilon_{k}=j\right\}-\mu^{k}E\left\{V_{k}|\varepsilon_{k}=j\right\}\\ = & \;\mu^{k+1}E\left\{V_{k+1}|\varepsilon_{k}=j\right\}-\mu^{k}E\left\{V_{k}|\varepsilon_{k}=j\right\}+\mu^{k}E\left\{V_{k}|\varepsilon_{k}=j\right\}-\mu^{k+1}E\left\{V_{k}|\varepsilon_{k}=j\right\}\\ = & \;\mu^{k+1}E\left\{V_{k+1}|\varepsilon_{k}=j\right\}-\mu^{k}E\left\{V_{k}|\varepsilon_{k}=j\right\}+\mu^{k}\left(1-\mu \right)E\left\{V_{k}|\varepsilon_{k}=j\right\}\\ \leq & \;-\mu^{k+1}\alpha E\left\{V_{k}|\varepsilon_{k}=j\right\}+\mu^{k+1}\zeta . \end{array} \end{array}$$

Hence, we have

(26) $$\begin{array}{c} \begin{array}{cc} \; & \;E\{\mu^{k+1}V_{k+1}-\mu^{k}V_{k}\}\\ \leq & \;-\mu^{k+1}\alpha E\left\{V_{k}\right\}+\mu^{k+1}\zeta -\mu^{k}\left(1-\mu \right)E\left\{V_{k}\right\}\\ = & \;-\mu^{k+1}\alpha E\left\{V_{k}\right\}-\mu^{k}\left(1-\mu \right)E\left\{V_{k}\right\}+\mu^{k+1}\zeta \\ = & \;\mu^{k}\left(\mu -1\right)E\left\{V_{k}\right\}-\mu^{k+1}\alpha E\left\{V_{k}\right\}+\mu^{k+1}\zeta \\ = & \;\mu^{k}\left(\mu -1-\mu \alpha \right)E\left\{V_{k}\right\}+\mu^{k+1}\zeta . \end{array} \end{array}$$

Letting $\mu =\mu_{0}=\frac{1}{1-\alpha }$ and summing up both sides of (26) from 0 to τ−1 with respect to *k*, i.e.,

(27) $$\begin{array}{c} \begin{array}{cc} E\left\{\mu_{0}^{\tau }V_{\tau }\right\}-E\left\{\mu_{0}^{\tau -1}V_{\tau -1}\right\} & \leq \mu_{0}^{\tau }\zeta ,\\ E\left\{\mu_{0}^{\tau -1}V_{\tau -1}\right\}-E\left\{\mu_{0}^{\tau -2}V_{\tau -2}\right\} & \leq \mu_{0}^{\tau -1}\zeta ,\\ \; & \;\;\vdots \\ E\left\{\mu_{0}^{2}V_{2}\right\}-E\left\{\mu_{0}^{1}V_{1}\right\} & \leq \mu_{0}^{2}\zeta ,\\ E\left\{\mu_{0}^{1}V_{1}\right\}-E\left\{\mu_{0}^{0}V_{0}\right\} & \leq \mu_{0}^{1}\zeta . \end{array} \end{array}$$

Then, we have

(28) $$\begin{array}{c} \begin{array}{cc} \; & E\left\{\mu_{0}^{\tau }V_{\tau }\right\}-E\left\{V_{0}\right\}\\ \leq & \mu_{0}^{\tau }\zeta +\mu_{0}^{\tau -1}\zeta +\cdots +\mu_{0}^{2}\zeta +\mu_{0}^{1}\zeta \\ \leq & \frac{\mu_{0}\left(1-\mu_{0}^{\tau }\right)}{1-\mu_{0}}\zeta , \end{array} \end{array}$$

and it follows that

(29) $$\begin{array}{cc} \; & E\{V_{k}\}\\ \leq & \mu_{0}^{-k}\left(E\left\{V_{0}\right\}+\frac{\mu_{0}\left(1-\mu_{0}^{k}\right)}{1-\mu_{0}}\zeta \right)\\ \leq & \mu_{0}^{-k}E\left\{V_{0}\right\}+\frac{\mu_{0}^{1-k}\left(1-\mu_{0}^{k}\right)}{1-\mu_{0}}\zeta \\ \leq & \mu_{0}^{-k}E\left\{V_{0}\right\}+\frac{\mu_{0}^{1-k}}{1-\mu_{0}}\zeta -\frac{\mu_{0}}{1-\mu_{0}}\zeta \\ \leq & \mu_{0}^{-k}E\left\{V_{0}\right\}-\frac{\mu_{0}^{1-k}}{\mu_{0}-1}\zeta +\frac{\mu_{0}}{\mu_{0}-1}\zeta \\ \leq & \mu_{0}^{-k}\left(E\left\{V_{0}\right\}-\frac{\mu_{0}}{\mu_{0}-1}\zeta \right)+\frac{\mu_{0}}{\mu_{0}-1}\zeta \\ \leq & {\left(1-\alpha \right)}^{k}\left(E\left\{V_{0}\right\}-\frac{\zeta }{\alpha }\right)+\frac{\zeta }{\alpha }. \end{array}$$

Then, it is easy to see that

(30) $$\begin{array}{c} \begin{array}{cc} \; & \;E\{∥e_{k}{∥}^{2}\}\\ \leq & \;\frac{1}{\overset{ˇ}{p}}E\left\{V_{k}\right\}\\ \leq & \;\frac{{\left(1-\alpha \right)}^{k}}{\overset{ˇ}{p}}\left(E\left\{V_{0}\right\}-\frac{\zeta }{\alpha }\right)+\frac{\zeta }{\alpha \overset{ˇ}{p}}\\ \leq & \;\frac{{\left(1-\alpha \right)}^{k}}{\overset{ˇ}{p}}E\left\{V_{0}\right\}+\frac{\zeta }{\alpha \overset{ˇ}{p}}\\ \leq & \;\frac{{\left(1-\alpha \right)}^{k}}{\overset{ˇ}{p}}\underset{-T\leq \theta \leq 0}{\sup}{E\{∥\iota \left(\theta \right)∥}^{2}\}+\frac{\zeta }{\alpha \overset{ˇ}{p}} \end{array} \end{array}$$

where $\overset{ˇ}{p}≜\min_{1\leq j\leq l}\{\lambda_{\min}\left\{P_{j}\}\right\}$. Hence, it can be concluded that the system (15) is EUBMS subject to the quantization error $\Delta_{k}$, the measurement noise $\nu_{k}$, and the process noise $\omega_{k}$. The proof is complete. □

**Remark** **5.** *Note that sometimes the covariances of the process and measurement noises might be unknown. A viable method is to design the desired filter based on certain “virtual” covariances, which are sufficiently large to “cover" the effects induced by the noises. The detailed information about such a method can be found in [47,48,49].*

**Remark** **6.** *Theorem 1 offers a sufficient condition to guarantee the EUB of the dynamics (15) in mean square under the effects of the RAP scheduling and uniform quantization. From (30), we can easily see that the ultimate bound of (15) is $\frac{\zeta }{\alpha \overset{ˇ}{p}}$.*

In what follows, it will be shown that the filter gain matrices $K_{j}$ (j=1,2,⋯,m) can be derived via solving a set of LMIs.

**Theorem** **2.** *Let a scalar 0<α<1 be given. Assume that there exist 2m+3 positive definite matrices $Q\in R^{\left(m+n)\times (m+n\right)}$, $H\in R^{n_{\omega }\times n_{\omega }}$, $T\in R^{n_{\nu }\times n_{\nu }}$, $Z_{j}\in R^{m\times (m+n)}$(j=1,2,⋯,m), $P_{j}\in R^{\left(m+n)\times (m+n\right)}$(j=1,2,⋯,m) and three positive scalars $\gamma_{\varsigma }$(ς=1,2,3), such that the following LMIs hold, for j=1,2,⋯,m:*

(31) $$\begin{array}{c} \begin{array}{c} {\overset{´}{\bar{\Lambda }}}^{j}=\left[\begin{array}{cccccc} {\overset{´}{\bar{\Lambda }}}_{11}^{j} & 0 & 0 & 0 & 0 & {\overset{´}{\bar{\Lambda }}}_{16}^{j}\\ {}* & {\overset{´}{\bar{\Lambda }}}_{22}^{j} & 0 & 0 & 0 & {\overset{´}{\bar{\Lambda }}}_{26}^{j}\\ {}* & * & {\overset{´}{\bar{\Lambda }}}_{33}^{j} & 0 & 0 & {\overset{´}{\bar{\Lambda }}}_{36}^{j}\\ {}* & * & * & {\overset{´}{\bar{\Lambda }}}_{44}^{j} & 0 & {\overset{´}{\bar{\Lambda }}}_{46}^{j}\\ {}* & * & * & * & {\overset{´}{\bar{\Lambda }}}_{55}^{j} & 0\\ {}* & * & * & * & * & {\overset{´}{\bar{\Lambda }}}_{66}^{j} \end{array}\right]<0 \end{array} \end{array}$$

*where*

$$\begin{array}{c} A_{331}^{j}≜\left[\begin{array}{ccc} I_{n_{\omega }\times n_{\omega }} & 0_{n_{\omega }\times n_{\nu }} & 0_{n_{\omega }\times m} \end{array}\right],\;\;A_{332}^{j}≜\left[\begin{array}{ccc} 0_{n_{\nu }\times n_{\omega }} & I_{n_{\nu }\times n_{\nu }} & 0_{n_{\nu }\times m} \end{array}\right],\;\;\\ A_{221}^{j}≜\left[\begin{array}{cc} I_{n\times n} & 0_{n\times m}\\ 0_{m\times n} & 0_{m\times m} \end{array}\right],\;\;A_{111}^{j}≜\left[\begin{array}{cc} a_{1}^{2}I_{n\times n} & 0_{n\times m}\\ 0_{m\times n} & 0_{m\times m} \end{array}\right],\;\;A_{222}^{j}≜\left[\begin{array}{cc} 0_{n\times n} & 0_{n\times m}\\ 0_{m\times n} & I_{m\times m} \end{array}\right],\\ {\overset{´}{\bar{\Lambda }}}_{16}^{j}≜{\tilde{A}}_{j}^{T}\sum_{i=1}^{m}p_{ji}P_{i}{-\bar{A}}_{j}^{T}Z_{j},\;\;A_{441}^{j}≜\left[\begin{array}{cc} a_{1}^{2}I_{n\times n} & 0_{n\times m}\\ 0_{m\times n} & 0_{m\times m} \end{array}\right],\;\;{\overset{´}{\bar{\Lambda }}}_{44}^{j}≜\gamma_{1}A_{441}^{j}-Q+\alpha Q,\\ A_{333}^{j}≜\left[\begin{array}{ccc} 0_{m\times n_{\omega }} & 0_{m\times n_{\nu }} & I_{m\times m} \end{array}\right],\;\;A_{112}^{j}≜\left[\begin{array}{cc} a_{2}^{2}I_{n\times n} & 0_{n\times m}\\ 0_{m\times n} & 0_{m\times m} \end{array}\right],\\ {\overset{´}{\bar{\Lambda }}}_{11}^{j}≜TQ-P_{j}+\gamma_{1}A_{111}^{j}+\gamma_{2}A_{112}^{j}+\alpha P_{j},\;\;{\overset{´}{\bar{\Lambda }}}_{22}^{j}≜-\gamma_{1}A_{221}^{j}-\gamma_{2}A_{222}^{j},\;\;\\ {\overset{´}{\bar{\Lambda }}}_{26}^{j}≜{\tilde{B}}_{j}^{T}\sum_{i=1}^{m}p_{ji}P_{i}{-\bar{B}}_{j}^{T}Z_{j},\;\;{\overset{´}{\bar{\Lambda }}}_{36}^{j}≜{\tilde{C}}_{j}^{T}\sum_{i=1}^{m}p_{ji}P_{i}{-\bar{C}}_{j}^{T}Z_{j},\;\;{\overset{´}{\bar{\Lambda }}}_{46}^{j}≜{\tilde{D}}_{j}^{T}\sum_{i=1}^{m}p_{ji}P_{i}{-\bar{D}}_{j}^{T}Z_{j},\\ {\overset{´}{\bar{\Lambda }}}_{3,3}^{j}≜-{A_{331}^{j}}^{T}HA_{331}^{j}-{A_{332}^{j}}^{T}TA_{332}^{j}-{A_{333}^{j}}^{T}\gamma_{3}A_{333}^{j},\;\;{\overset{´}{\bar{\Lambda }}}_{66}^{j}≜-\sum_{i=1}^{m}p_{ji}P_{i},\\ {\overset{´}{\bar{\Lambda }}}_{55}^{j}≜\mathrm{diag}\left\{-Q+2\alpha Q,-Q+3\alpha Q,\cdots ,-Q+T\alpha Q\right\}. \end{array}$$

*Then, the filtering error dynamics (15) is EUBMS. Moreover, the filter gain matrices can be calculated by $K_{j}={\left(\sum_{i=1}^{m}p_{ji}P_{i}\right)}^{-1}Z_{j}^{T}$.*

**Proof** **of** **Theorem** **2.** According to the well-known Schur Complement lemma [50], $\Lambda_{j}$ can be rewritten as

(32) $$\begin{array}{cc} \Lambda_{j}= & {\vec{\Lambda }}_{j}+\left[\begin{array}{c} {\vec{A}}_{j}^{T}\\ {\vec{B}}_{j}^{T}\\ {\vec{C}}_{j}^{T}\\ {\vec{D}}_{j}^{T}\\ 0 \end{array}\right]\sum_{i=1}^{m}p_{ji}P_{i}\left[\begin{array}{ccccc} {\vec{A}}_{j} & {\vec{B}}_{j} & {\vec{C}}_{j} & {\vec{D}}_{j} & 0 \end{array}\right]\\ = & {\vec{\Lambda }}_{j}+\left[\begin{array}{c} {\vec{A}}_{j}^{T}\\ {\vec{B}}_{j}^{T}\\ {\vec{C}}_{j}^{T}\\ {\vec{D}}_{j}^{T}\\ 0 \end{array}\right]\sum_{i=1}^{m}p_{ji}P_{i}P_{i}^{-1}P_{i}\left[\begin{array}{ccccc} {\vec{A}}_{j} & {\vec{B}}_{j} & {\vec{C}}_{j} & {\vec{D}}_{j} & 0 \end{array}\right]\\ = & {\vec{\Lambda }}_{j}+\left[\begin{array}{c} {\vec{A}}_{j}^{T}\\ {\vec{B}}_{j}^{T}\\ {\vec{C}}_{j}^{T}\\ {\vec{D}}_{j}^{T}\\ 0 \end{array}\right]\sum_{i=1}^{m}p_{ji}P_{i}^{T}P_{i}^{-1}P_{i}\left[\begin{array}{ccccc} {\vec{A}}_{j} & {\vec{B}}_{j} & {\vec{C}}_{j} & {\vec{D}}_{j} & 0 \end{array}\right]\\ = & {\vec{\Lambda }}_{j}+\left[\begin{array}{c} {\vec{A}}_{j}^{T}P_{i}^{T}\\ {\vec{B}}_{j}^{T}P_{i}^{T}\\ {\vec{C}}_{j}^{T}P_{i}^{T}\\ {\vec{D}}_{j}^{T}P_{i}^{T}\\ 0 \end{array}\right]\sum_{i=1}^{m}p_{ji}P_{i}^{-1}\left[\begin{array}{ccccc} P_{i}{\vec{A}}_{j} & P_{i}{\vec{B}}_{j} & P_{i}{\vec{C}}_{j} & P_{i}{\vec{D}}_{j} & 0 \end{array}\right] \end{array}$$

where

$$\begin{array}{c} {\vec{\Lambda }}_{j}≜\left[\begin{array}{ccccc} {\overset{´}{\bar{\Lambda }}}_{11}^{j} & 0 & 0 & 0 & 0\\ {}* & {\overset{´}{\bar{\Lambda }}}_{22}^{j} & 0 & 0 & 0\\ {}* & * & {\overset{´}{\bar{\Lambda }}}_{33}^{j} & 0 & 0\\ {}* & * & * & {\overset{´}{\bar{\Lambda }}}_{44}^{j} & 0\\ {}* & * & * & * & {\overset{´}{\bar{\Lambda }}}_{55}^{i} \end{array}\right]. \end{array}$$

Then, it is easy to see from (31) that $\Lambda_{j}<0$. Hence, it follows from Theorem 2 that the filter gain matrices $K_{j}$ can be given by resorting to the solution to the proposed LMIs. □

**Remark** **7.** *So far, we have addressed the ultimately bounded filtering problem for a class of time-delay nonlinear stochastic systems subject to the RAP scheduling and the UQEs. The upper bound of the filtering error has been expressed explicitly. We have presented sufficient conditions under which the desired ultimately bounded filter exists by means of certain matrix inequalities. In addition, the filter gain matrices $K_{j}$ have been designed in Theorem 2 by solving a set of LMIs.*

**Remark** **8.** *It is worth mentioning that our main results are different from existing ones in the following two aspects: (1) the proposed scheme is one of the first few attempts to address the ultimately bounded filtering problem under RAP and UQEs, which better caters for the engineering practice; and (2) the established theoretical framework of the networked systems is quite general, which takes both RAP and uniform quantization into account.*

## 4. Illustrative Examples

### 4.1. Example 1

Consider the following nonlinear stochastic time-delayed system:

$$\begin{array}{c} \left\{\begin{array}{cc} x_{1,k+1}= & \;0.52x_{1,k}+0.62x_{2,k}+0.64\sin\left(x_{1,k}\right)+0.02\omega_{k}\\ x_{2,k+1}= & \;0.56x_{1,k}+0.52x_{2,k}+0.32\sin\left(x_{2,k-2}\right)+0.02\omega_{k}\\ x_{3,k+1}= & \;0.5x_{3,k}+0.02\omega_{k}\\ y_{1,k}= & \;0.28x_{1,k}+0.27x_{2,k}+x_{3,k}+0.64\sin\left(x_{1,k}\right)+0.01\nu_{k}\\ y_{2,k}= & \;0.15x_{1,k}+0.19x_{2,k}+0.32\sin\left(x_{2,k}\right)+0.01\nu_{k}. \end{array}\right. \end{array}$$

From (1) and (11), we can obtain that

$$\begin{array}{cc} \; & \;A=\left[\begin{array}{ccc} 0.52 & 0.62 & 0\\ 0.56 & 0.52 & 0\\ 0 & 0 & 0.5 \end{array}\right],\;B=0,\;T=2,\;C=\left[\begin{array}{ccc} 0.28 & 0.27 & 1\\ 0.15 & 0.19 & 0 \end{array}\right],\;D_{1}={\left[\begin{array}{ccc} 0.02 & 0.02 & 0.02 \end{array}\right]}^{T},\\ \; & \;D_{2}={\left[\begin{array}{cc} 0.01 & 0.01 \end{array}\right]}^{T},\;a_{1}=0.1,\;a_{2}=0.1,\;\tilde{h}\left(x_{k}\right)=\left[\begin{array}{c} 0.64\sin(x_{1,k})\\ 0.32\sin(x_{2,k}) \end{array}\right],\\ \; & \;\tilde{f}\left(x_{k},x_{k-T}\right)=\left[\begin{array}{c} 0.64\sin(x_{1,k})\\ 0.32\sin(x_{2,k-2})\\ 0 \end{array}\right]. \end{array}$$

The matrices $\bar{L}$ and $\bar{Y}$ are given by 0.8 and 0.9, respectively. For the above system, assume that there are two sensors connected to the communication channel and the transition probability matrix of the RAP is given by

$$\begin{array}{cc} \Pi & =\left[\begin{array}{cc} 0.3 & 0.7\\ 0.2 & 0.8 \end{array}\right]. \end{array}$$

Set the parameter α as α=0.01. Then, by solving the inequalities presented in Theorem 2, the desired filter gain matrices $K_{j}$ can be calculated directly as follows:

$$\begin{array}{c} K_{1}=\left[\begin{array}{cc} 1.2677 & -6.4683\\ 1.2039 & 1.0281\\ 0.1225 & -5.6653\\ 1.0000 & -5.1454\\ 2.9017 & 1.0000 \end{array}\right],\;\;\;K_{2}=\left[\begin{array}{cc} -1.9956 & 2.5173\\ -7.1714 & 2.3810\\ 2.9402 & -0.0027\\ 1.0000 & 6.2394\\ 2.1537 & 1.0000 \end{array}\right]. \end{array}$$

Let the initial state be

$$\begin{array}{cc} {\bar{y}}_{0}= & \;\left[\begin{array}{c} 0\\ 0 \end{array}\right],\;{\bar{y}}_{1}=\left[\begin{array}{c} 0.1\\ 0.1 \end{array}\right],\;{\hat{y}}_{0}=\left[\begin{array}{c} 0\\ 0 \end{array}\right],\;{\hat{y}}_{1}=\left[\begin{array}{c} 0.5\\ 0.6 \end{array}\right],\;{\hat{x}}_{2}=\left[\begin{array}{c} -2\\ 10\\ 2 \end{array}\right]\\ x_{0}= & \;\left[\begin{array}{c} 5\\ -3\\ 0.6 \end{array}\right],\;x_{1}=\left[\begin{array}{c} 0\\ 0\\ 0 \end{array}\right],\;x_{2}=\left[\begin{array}{c} 0.1\\ 0.1\\ 0.1 \end{array}\right],\;{\hat{x}}_{0}=\left[\begin{array}{c} 0\\ 0\\ 0 \end{array}\right],\;{\hat{x}}_{1}=\left[\begin{array}{c} 0\\ 0\\ 0 \end{array}\right]. \end{array}$$

**Figure 2:**
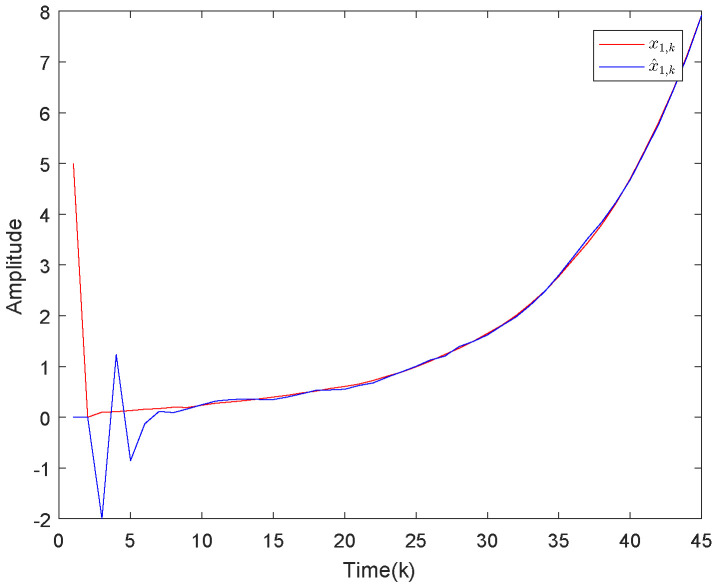
The state evolutions of $x_{1,k}$ and corresponding estimates ${\hat{x}}_{1,k}$.

**Figure 3:**
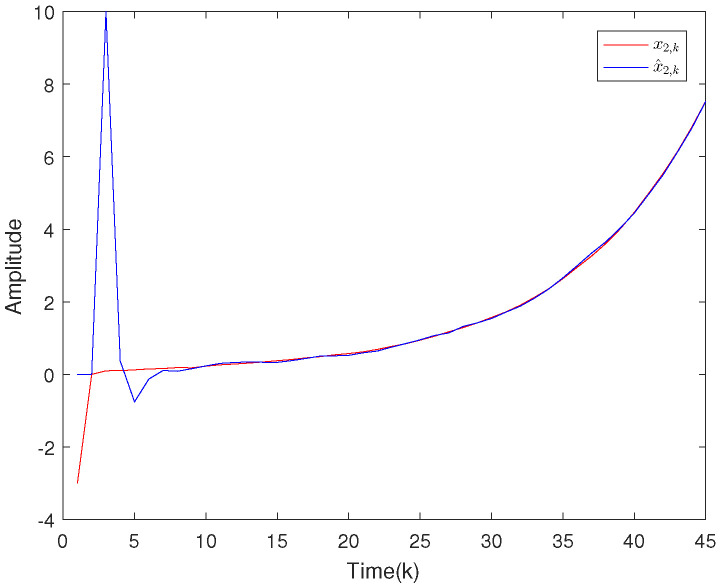
The state evolutions of $x_{2,k}$ and corresponding estimates ${\hat{x}}_{2,k}$.

**Figure 4:**
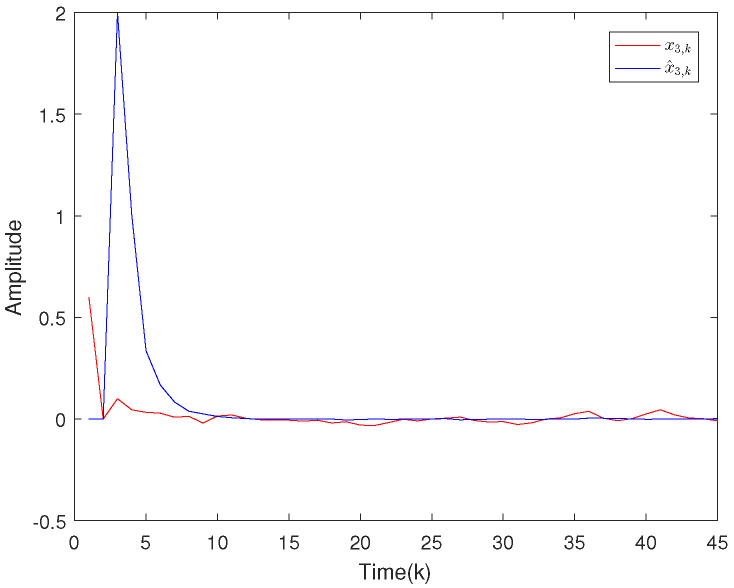
The state evolutions of $x_{3,k}$ and corresponding estimates ${\hat{x}}_{3,k}$.

**Figure 5:**
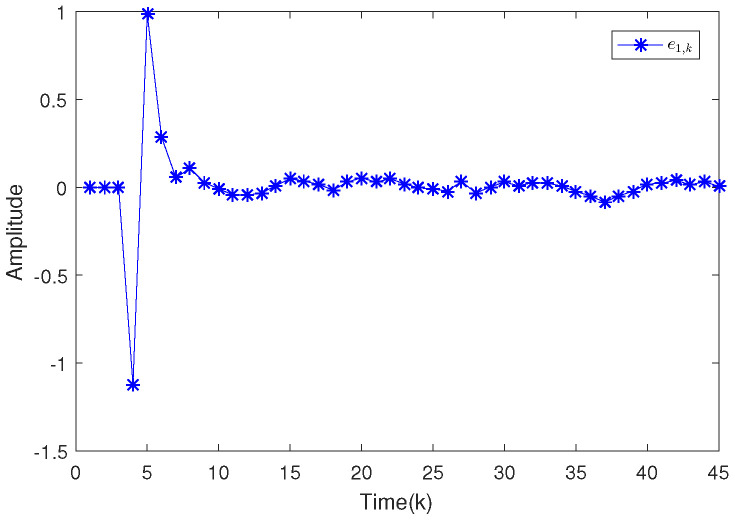
Filtering error $e_{1,k}$ under the RAP scheduling.

**Figure 6:**
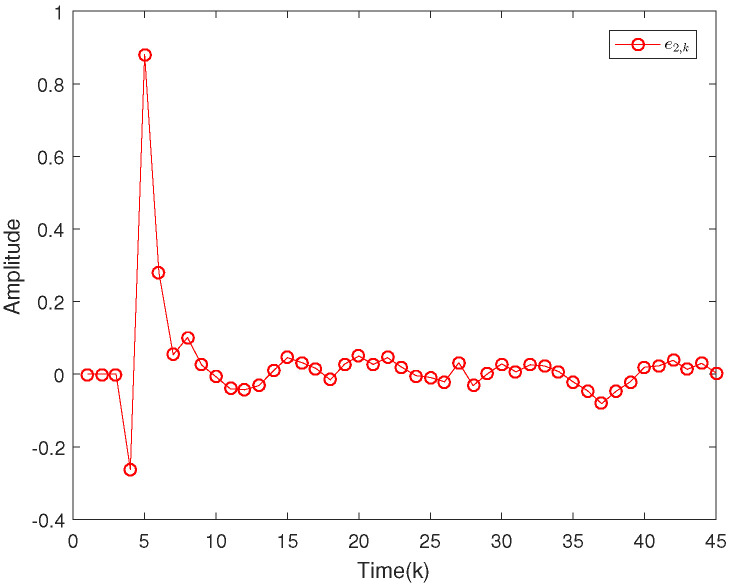
Filtering error $e_{2,k}$ under the RAP scheduling.

**Figure 7:**
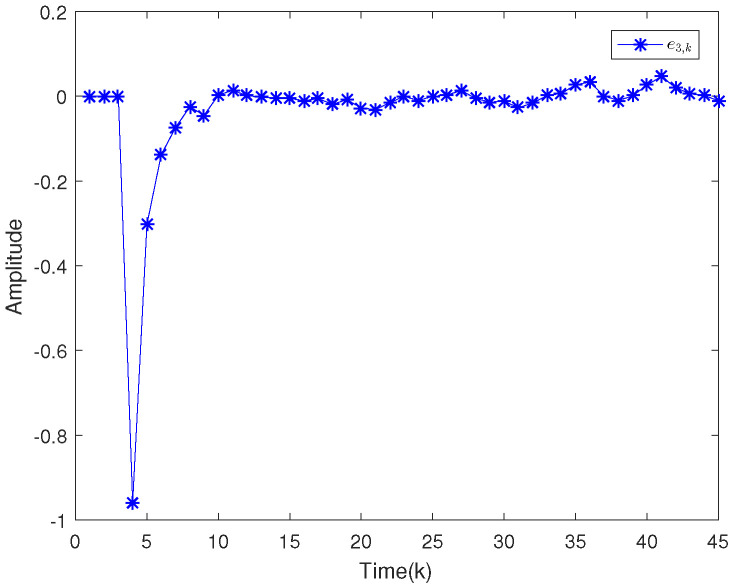
Filtering error $e_{3,k}$ under the RAP scheduling.

**Figure 8:**
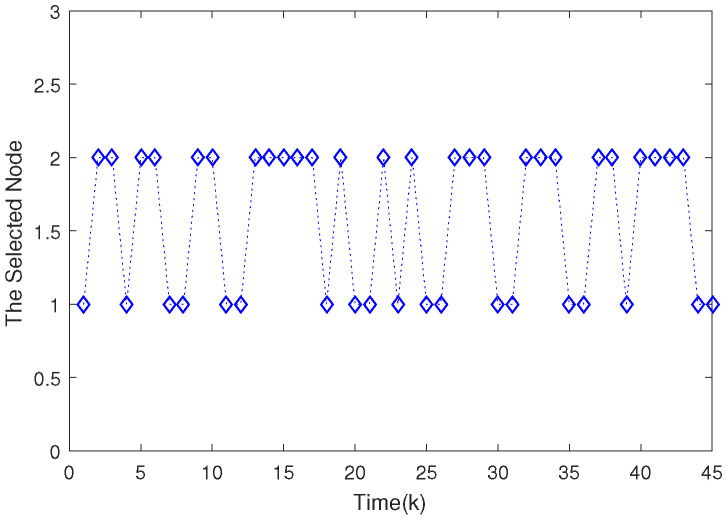
The evolutions of selected node under the RAP scheduling.

Based on the system model, the proposed filter structure as well as the derived filter gains, numerical simulation results are given in Figure 2, Figure 3, Figure 4, Figure 5, Figure 6, Figure 7 and Figure 8. Among them, Figure 2, Figure 3 and Figure 4 show the state trajectories and their corresponding estimates for $x_{1,k}$, $x_{2,k}$ and $x_{3,k}$, respectively. The filtering error $e_{1,k}$ under the RAP scheduling is depicted in Figure 5. Figure 6 and Figure 7 depict the filtering errors $e_{2,k}$ and $e_{3,k}$ under the RAP scheduling, respectively. In Figure 8, the access situation of two sensor nodes under the RAP scheduling is exhibited, from which we can clearly see the random selection feature of the RAP. On the other hand, it is obvious that the system under consideration is indeed unstable. The simulation results have verified that the designed ultimately bounded filter performs very well.

### 4.2. Example 2

In order to further verify the effectiveness of the proposed filtering method, we consider the following time-delayed nonlinear stochastic system:

$$\begin{array}{c} \left\{\begin{array}{cc} x_{1,k+1}= & \;0.52x_{1,k}+0.62x_{2,k}+0.32\sin\left(x_{1,k}\right)+0.02\omega_{k}\\ x_{2,k+1}= & \;0.6x_{1,k}+0.52x_{2,k}+0.96\sin\left(x_{2,k-2}\right)+0.02\omega_{k}\\ y_{1,k}= & \;0.28x_{1,k}+0.27x_{2,k}+0.64\sin\left(x_{1,k}\right)+0.01\nu_{k}\\ y_{2,k}= & \;0.15x_{1,k}+0.19x_{2,k}+0.01\nu_{k}. \end{array}\right. \end{array}$$

From (1) and (11), we can see that

$$\begin{array}{cc} \; & \;A=\left[\begin{array}{cc} 0.52 & 0.62\\ 0.6 & 0.52 \end{array}\right],\;B=0,\;T=2,\;C=\left[\begin{array}{cc} 0.28 & 0.27\\ 0.15 & 0.19 \end{array}\right],\;D_{1}={\left[\begin{array}{cc} 0.02 & 0.02 \end{array}\right]}^{T},\\ \; & \;D_{2}={\left[\begin{array}{cc} 0.01 & 0.01 \end{array}\right]}^{T},\;a_{1}=0.1,\;a_{2}=0.1,\;\tilde{h}\left(x_{k}\right)=\left[\begin{array}{c} 0.64\sin(x_{1,k})\\ 0 \end{array}\right],\\ \; & \;\tilde{f}\left(x_{k},x_{k-T}\right)=\left[\begin{array}{c} 0.32\sin(x_{1,k})\\ 0.96\sin(x_{2,k-2}) \end{array}\right]. \end{array}$$

The matrices $\bar{L}$ and $\bar{Y}$ are set to be 0.8 and 0.9, respectively. The transition probability matrix of the RAP is selected as

$$\begin{array}{cc} \Pi & =\left[\begin{array}{cc} 0.4 & 0.6\\ 0.2 & 0.8 \end{array}\right]. \end{array}$$

The parameter α is set to be 0.3. Then, the filter gains $K_{j}$ are obtained by using Theorem 2:

$$\begin{array}{c} K_{1}=\left[\begin{array}{cc} 2.0210 & 3.2909\\ 1.9908 & -1.1649\\ 1.0000 & -3.5153\\ -1.4609 & 1.0000 \end{array}\right],\;\;\;K_{2}=\left[\begin{array}{cc} -8.2250 & 3.0315\\ -1.0058 & 2.9526\\ 1.0000 & 3.8047\\ 1.3884 & 1.0000 \end{array}\right]. \end{array}$$

The initial states are chosen as:

$$\begin{array}{cc} {\bar{y}}_{0}= & \;\left[\begin{array}{c} 0\\ 0 \end{array}\right],\;{\bar{y}}_{1}=\left[\begin{array}{c} 0.1\\ 0.1 \end{array}\right],\;{\hat{y}}_{0}=\left[\begin{array}{c} 0\\ 0 \end{array}\right],\;{\hat{y}}_{1}=\left[\begin{array}{c} 0.5\\ 0.6 \end{array}\right],\;{\hat{x}}_{2}=\left[\begin{array}{c} -2\\ 10 \end{array}\right]\\ x_{0}= & \;\left[\begin{array}{c} 5\\ -3 \end{array}\right],\;x_{1}=\left[\begin{array}{c} 0\\ 0 \end{array}\right],\;x_{2}=\left[\begin{array}{c} 0.1\\ 0.1 \end{array}\right],\;{\hat{x}}_{0}=\left[\begin{array}{c} 0\\ 0 \end{array}\right],\;{\hat{x}}_{1}=\left[\begin{array}{c} 0\\ 0 \end{array}\right]. \end{array}$$

**Figure 9:**
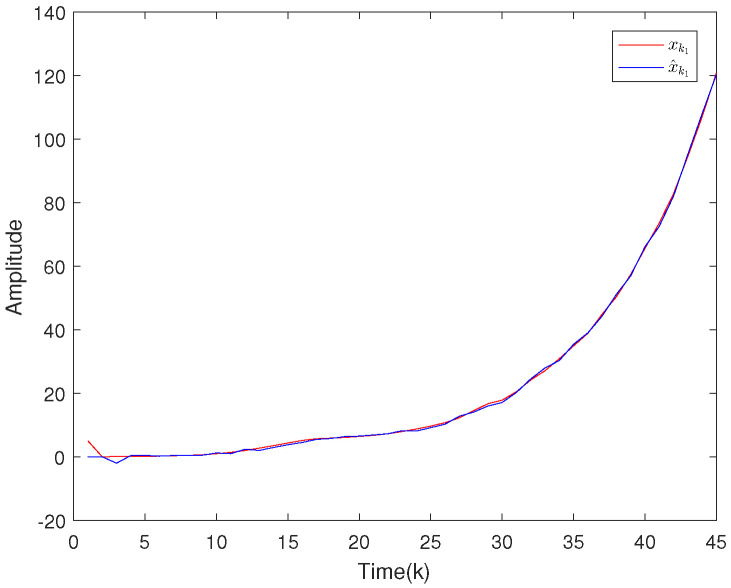
The state evolutions of $x_{1,k}$ and corresponding estimates ${\hat{x}}_{1,k}$.

**Figure 10:**
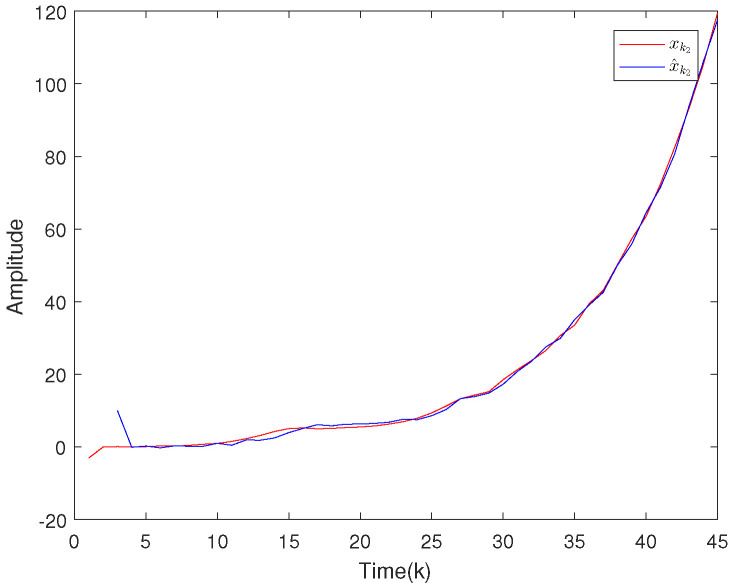
The state evolutions of $x_{2,k}$ and corresponding estimates ${\hat{x}}_{2,k}$.

**Figure 11:**
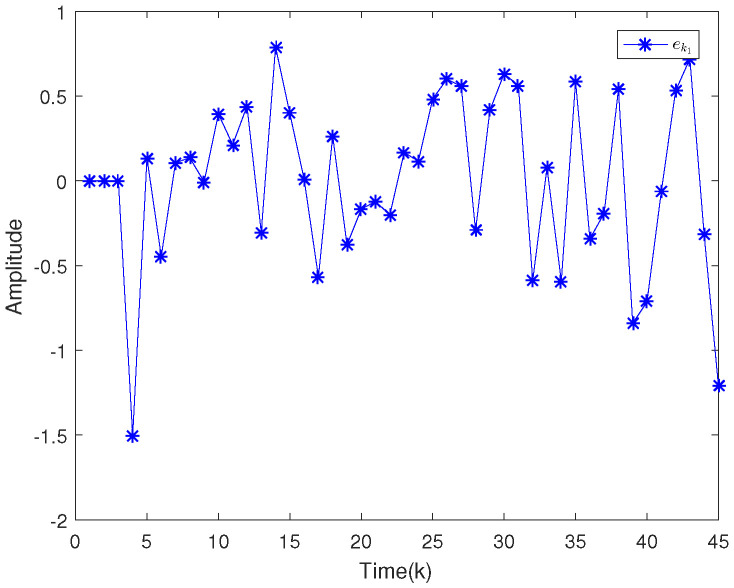
Filtering error $e_{1,k}$ under the RAP scheduling.

**Figure 12:**
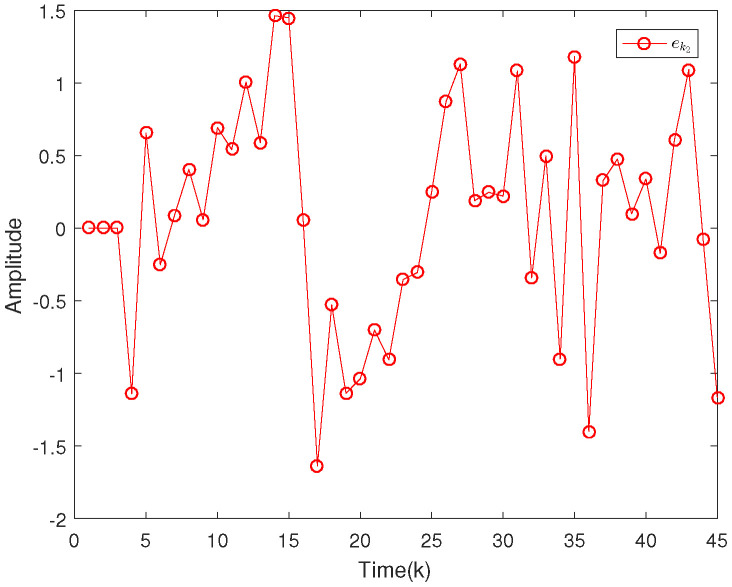
Filtering error $e_{2,k}$ under the RAP scheduling.

**Figure 13:**
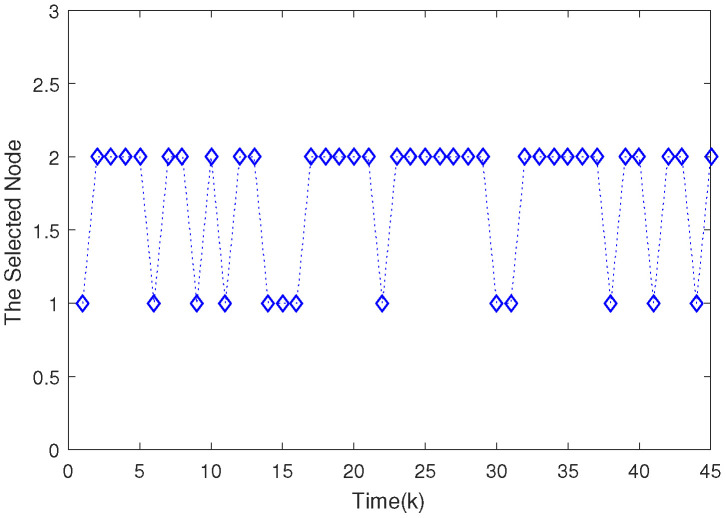
The evolutions of selected node under the RAP scheduling.

The simulation results are given in Figure 9, Figure 10, Figure 11, Figure 12 and Figure 13. Among them, the state trajectories and their corresponding estimates for $x_{1,k}$ and $x_{2,k}$ are depicted in Figure 9 and Figure 10, respectively. Figure 11 and Figure 12 show the filtering error $e_{1,k}$ and $e_{2,k}$ under the RAP scheduling. Figure 13 exhibits the access situation of the sensors.

Next, let us consider the effects of the noise covariances on the ultimate bound. The simulation results are given in Table 1, from which we can easily see the large noise covariances would lead to a large ultimate bound of the filtering error.

## 5. Conclusions

In this paper, the ultimately bounded filtering problem has been studied for a class of time-delay nonlinear stochastic systems with RAP scheduling and UQEs. The scheduling behavior of the so-called RAP has been modeled by a discrete-time homogeneous Markov chain with known transition probability matrix. A novel and easy-to-implement ultimately bounded filter has been presented to reconstruct the real state variables under the pre-defined performance index, and the desired filter gains have been derived by solving a set of LMIs. Finally, two simulation examples have been exploited to verify the validity of the proposed filtering scheme. 

## Figures and Tables

**Table 1:** The effects of the noise covariances on the ultimate bound.

$E\{\omega_{k}\omega_{k}^{T}\}$	0.64	0.16	0.0064
$E\{\nu_{k}\nu_{k}^{T}\}$	0.81	0.225	0.0081
Ultimate bound	163.9338	43.5281	1.6394

## Abbreviations

The following abbreviations are used in this manuscript:

RAP	random access protocol
UQEs	uniform quantization effcets
EUBMS	exponentially ultimately bounded in mean square
LMIs	linear matrix inequalities
EUB	exponential ultimate boundedness
ZOH	zero-order holder
